# Doxorubicin-loaded cholic acid-polyethyleneimine micelles for targeted delivery of antitumor drugs: synthesis, characterization, and evaluation of their *in vitro* cytotoxicity

**DOI:** 10.1186/1556-276X-7-687

**Published:** 2012-12-28

**Authors:** Muhammad Wahab Amjad, Mohd Cairul Iqbal Mohd Amin, Haliza Katas, Adeel Masood Butt

**Affiliations:** 1 Centre for Drug Delivery Research, Faculty of Pharmacy, Universiti Kebangsaan Malaysia, Jalan Raja Muda Abdul Aziz, Kuala Lumpur, 50300, Malaysia

**Keywords:** Micelles, Nanoparticles, Cholic acid, Polyethyleneimine, Doxorubicin

## Abstract

Doxorubicin-loaded micelles were prepared from a copolymer comprising cholic acid (CA) and polyethyleneimine (PEI) for the delivery of antitumor drugs. The CA-PEI copolymer was synthesized via pairing mediated by *N*,*N*’-dicyclohexylcarbodiimide and *N*-hydroxysuccinimide using dichloromethane as a solvent. Fourier transform infrared and nuclear magnetic resonance analyses were performed to verify the formation of an amide linkage between CA and PEI and doxorubicin localization into the copolymer. Dynamic light scattering and transmission electron microscopy studies revealed that the copolymer could self-assemble into micelles with a spherical morphology and an average diameter of <200 nm. The CA-PEI copolymer was also characterized by X-ray diffraction and differential scanning calorimetry. Doxorubicin-loaded micelles were prepared by dialysis method. A drug release study showed reduced drug release with escalating drug content. In a cytotoxicity assay using human colorectal adenocarcinoma (DLD-1) cells, the doxorubicin-loaded CA-PEI micelles exhibited better antitumor activity than that shown by doxorubicin. This is the first study on CA-PEI micelles as doxorubicin carriers, and this study demonstrated that they are promising candidates as carriers for sustained targeted antitumor drug delivery system.

## Background

Several therapeutic anticancer drugs, although pharmacologically effective in cancer treatment, are restricted in their clinical applications because of their severe toxicity [1]. The severe toxicity is usually due to the lipid solubility of most of the anticancer drugs (>70%) and the therapeutic doses that are often very high [2]. Doxorubicin is one of the most successful drugs for targeting a broad range of cancers. Nevertheless, its clinical use is hindered by its side effects, which include cardiotoxicity and acquired drug resistance. To overcome these complications, researchers have placed an emphasis on developing nanoscale anticancer drug carriers for improving therapeutic efficacy in addition to reducing unwanted side effects [3].

Polymeric micelles self-assembled from amphiphilic copolymers have gained much interest for use in targeted anticancer drug delivery since they have a number of physico- and bio-chemical advantages over other types of nanocarriers. Polymeric micelles are virus-sized with a core-shell structure having a hydrophobic core and a hydrophilic shell and, more significantly, inherent stealth. Polymeric micelles seem ideal for the targeted and controlled delivery of hydrophobic anticancer drugs, including paclitaxel and doxorubicin [4], in that they significantly increase their water solubility, extend their circulation time, passively target tumor tissues [5], increase their bioavailability, have tremendous biocompatibility, and are degradable *in vivo* into nontoxic products. Several types of polymer blocks can be used to form micelles, of which the most studied include poly(α-hydroxy esters) [6] (such as polylactide [7], polyglycolide [8], and poly(ε-caprolactone) [9]), polyether [10], hydrotrophic polymers [11], and poly(amino acids) [12]. Several attempts have been made to formulate stable polymeric micelles with new surfactant combinations to achieve ideal drug delivery *in vitro* as well as *in vivo*.

Cholic acid (CA), a bile acid, is an amphiphilic steroid molecule naturally synthesized from cholesterol, which organizes into micelles above the critical micelle concentration (CMC). Bile acids, together with the phospholipids, vary the permeability of cell membranes [13]. Some bile acids form hydrogen-bonded aggregates with some drugs, which may lead to alterations in drug bioavailability [14]. Polyethyleneimine (PEI) is a cationic synthetic vector mainly used for gene delivery owing to its high nucleic acid condensing potential, ability to escape endosomes [15], nuclear localization capability [16], and promising transfection efficacy both *in vitro* and *in vivo*[15].

We synthesized doxorubicin-loaded cholic acid-polyethyleneimine (CA-PEI) micelles as an antitumor drug delivery system. The antitumor activity of the doxorubicin-loaded CA-PEI micelles was then tested using human colorectal adenocarcinoma (DLD-1) cells.

## Methods

### Materials

CA, PEI (average molecular weight (MW) approximately 1,300), *N*,*N*’-dicyclohexylcarbodiimide (DCC), *N*-hydroxysuccinimide (NHS), hydrochloric acid (HCl), triethylamine, tetrahydrofuran, and dichloromethane were purchased from Sigma-Aldrich (St. Louis, MO, USA). Doxorubicin was purchased from Calbiochem (Merck KGaA, Darmstadt, Germany). The Spectra/Por™ dialysis membrane (MW cutoff (MWCO) = 1,000 g/mol) was purchased from Spectrum Labs (Rancho Dominguez, CA, USA).

### Synthesis of the CA-PEI copolymer

**Figure 1:**
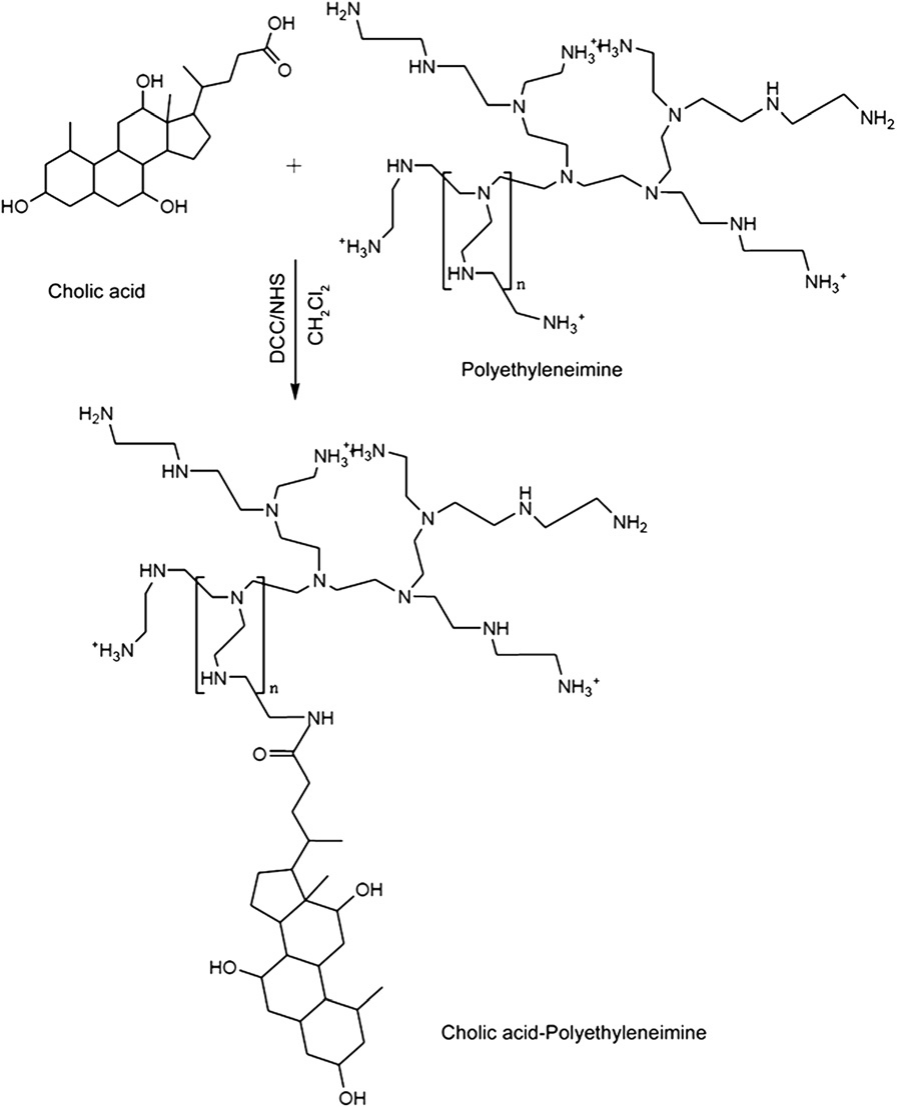
Schematic representation of carbodiimide-mediated coupling of CA-PEI.

The side-chain carboxyl group at the C-24 position in CA was conjugated to the terminal amine group of PEI. This was carried out by dissolving CA in tetrahydrofuran and activating it with DCC and NHS at 25°C for 8 h. CA was then precipitated in ice-cold *n*-hexane and dried in an oven at 40°C for 2 h. The activated CA was then conjugated to the primary amine group of PEI by incubating for 15 h in dichloromethane (Figure 1) using CA-PEI molar ratios of 1:1, 1:2, 1:4, 3:1, and 4:1.

The resulting conjugates were dried using a rotary evaporator and dissolved in dilute HCl followed by precipitation with cold acetone. Finally, they were dissolved in deionized water, filtered, and freeze-dried.

### Analysis of the conjugates

To assess their functional groups, drug-loaded and blank conjugates were characterized using a Fourier trans-form infrared (FTIR) spectrophotometer (Spectrum 100, PerkinElmer, Waltham, MA, USA) using the potassium bromide (KBr) disc method. For each sample, 16 scans were obtained at a resolution of 4 cm^−1^ in the range of 4,000 to 700 cm^−1^. Further characterization of the conjugates was also performed using nuclear magnetic resonance (NMR) spectroscopy (Bruker Avance III, FT-NMR 600 MHz with cryoprobe, Germany). The CMCs of the micelles were determined using the dynamic light scattering method (Zetasizer Nano ZS, Malvern Instruments, Malvern, Worcestershire, UK) at 37°C with a scattering angle of 90°. The alterations in light intensity were recorded, and a graph was plotted for the molar concentrations of the samples versus the mean intensity. A sharp increase in the intensity signified the formation of micelles. Samples for morphological investigations were prepared by air-drying a drop of the micellar suspension on a carbon-coated formvar film on a 400-mesh copper grid. The morphology of the micelles was then visualized by transmission electron microscopy (TEM; Tecnai™ Spirit, FEI, Eindhoven, The Netherlands) at 220 kV and under various magnifications. The conjugates were observed under a light microscope (FluoView FV1000, Olympus, Tokyo, Japan). The X-ray diffraction (XRD) patterns of the CA-PEI conjugates were analyzed with an X-ray diffractometer (D8 ADVANCE, Cu K*α* = 1.54184 Å, Bruker, WI, USA). The thermal behavior of the conjugates was investigated by differential scanning calo-rimetry (DSC) (Diamond DSC, PerkinElmer, Waltham, MA, USA).

### Preparation of the doxorubicin-loaded CA-PEI micelles

Doxorubicin hydrochloride (2.5 mg) was dissolved in 2 mL chloroform and mixed with 2 μL of triethylamine. CA-PEI copolymers of different molar ratios (1:1, 1:2, 1:4, 3:1, and 4:1) were dissolved in 2 mL methanol. The doxorubicin and CA-PEI copolymer solutions were mixed in a glass vial and kept in the dark for 24 h. The solution was then poured drop by drop into deionized water (20 mL) under ultrasonic agitation using a sonifier (Branson Ultrasonics Co., Danbury, CT, USA) at a power level of 3 for 10 min. The organic solvents namely chloroform and methanol were then completely removed by vacuum distillation using a rotary evaporator. The doxorubicin-loaded micelle solution was then dialyzed against 1 L of deionized water for 24 h at 20°C using a cellulose membrane bag (MWCO = 1,000) to remove unloaded doxorubicin. The deionized water was substituted every 2 h for the first 12 h and then every 6 h. Immediately after this, the product was freeze-dried. The extent of doxorubicin loaded into the micelles was determined from a calibration curve of pure doxorubicin. Freeze-dried doxorubicin-loaded micelles were dissolved in 4 mL of a DMSO and methanol mixture (1:1), and the absorbance was measured at 480 nm using a UV-1601 spectrophotometer (Shimadzu Corp., Kyoto, Japan).

The drug loading content (DLC) is defined as the ratio of mass of the drug encapsulated within the micelles to the total mass of drug-loaded micelles, while the entrapment efficiency (EE) is the ratio of mass of drug loaded into the micelles to the mass of drug initially added. The DLC and EE were calculated according to the following equations:

(1) $$\begin{array}{l} \text{DLC}\left(\text{wt}.\%\right)=\text{mass}\;\text{of}\;\text{drug}\;\text{encapsulated}\;\text{in}\;\text{micelles}/\\ \;\text{mass}\;\text{of}\;\text{drug}-\text{loaded}\;\text{micelles}\times 100 \end{array}$$

(2) $$\begin{array}{l} \text{EE}\left(\%\right)=\text{mass}\;\text{of}\;\text{drug}\;\text{loaded}\;\text{in}\;\text{micelles}/\\ \;\text{mass}\;\text{of}\;\text{drug}\;\text{initially}\;\text{added}\times 100 \end{array}$$

### *In vitro* drug release study

The drug release experiment was carried out *in vitro*. A doxorubicin-loaded micelle solution previously prepared by dialysis was used for release analysis. This solution was introduced into the dialysis membrane. Subsequently, the dialysis membrane was placed in a 200-mL beaker with 100 mL of phosphate-buffered saline (PBS). This beaker was placed on a magnetic stirrer with a stirring speed of 100 rpm at 37°C. At suitable intervals, 3 mL samples were taken from the release medium and an equivalent volume of fresh medium was added. The concentration of doxorubicin in each sample was measured by ultraviolet–visible spectrophotometry at 480 nm.

### Cytotoxicity analysis

Human colorectal adenocarcinoma (DLD-1) and Chinese hamster lung fibroblast (V79) cell lines were obtained from the American Type Culture Collection (Manassas, VA, USA). DLD-1 cells were cultured and maintained in Roswell Park Memorial Institute-1640 (RPMI-1640) medium, whereas V79 cells were cultured in Dulbecco’s modified Eagle’s medium (DMEM). Both cell lines were supplemented with 10% FBS and 1% penicillin-streptomycin and maintained at 37°C in a humidified 5% CO_2_/95% air atmosphere.

The impact of the blank micelles on cell viability was assessed using V79 cells. Cultured cells maintained in DMEM were seeded in 96-well culture plates at 4 × 10^4^ cells per well and incubated for 24 h. The cells were then treated with increasing concentrations of blank micelles ranging from 31.25 to 500 μg · mL^−1^ and incubated for an additional 24 h at 37°C in a 5% CO_2_/95% air atmosphere. Next, 20 μL of Alamar Blue® (Invitrogen, Carlsbad, CA, USA) was introduced to every well, and the cells were incubated for a further 4 h. The absorbance of each sample was measured at 570 nm with a microplate reader (Varioskan Flash, Thermo Scientific, Waltham, MA, USA). Cell viability was determined using the following equation:

(3) $$\text{Cell}\;\text{viability}\left(\%\right)=A_{570}\text{of}\;\text{treated}\;\text{cells}/A_{570}\text{of}\;\text{control}\;\text{cells}\times 100$$

The cytotoxicity of the doxorubicin-loaded micelles was determined using the Alamar blue assay. DLD-1 cells were seeded in 96-well culture plates at 2 × 10^4^ cells per well and incubated for 48 h at 37°C in 5% CO_2_/95% air atmosphere. After the medium was removed, the cells were treated with 200 μL of 50, 25, 12.5, 6.25, 1.56, 0.19, and 0.09 μg · mL^−1^ of free doxorubicin and doxorubicin-loaded micelles, respectively. After 24-h incubation, Alamar blue solution was added to each well, and the incubation was continued for 4 h. The absorbance of each sample at 570 nm (A_570_) was measured with a microplate reader. Cell viability was determined using the following equation:

(4) $$\text{Percentage}\;\text{inhibition}\left(\%\right)=100-\text{cell}\;\text{viability}\left(\%\right)$$

## Results and discussion

### Formation and characterization of the CA-PEI micelles

The facially amphipathic CA was introduced into PEI to prepare stable CA-PEI micelles as carriers for the delivery of doxorubicin. The CA terminal carboxyl group that was principally activated using DCC/NHS chemistry was conjugated to the PEI amine group via an amide linkage to obtain the CA-PEI conjugate (Figure 1).

**Figure 2:**
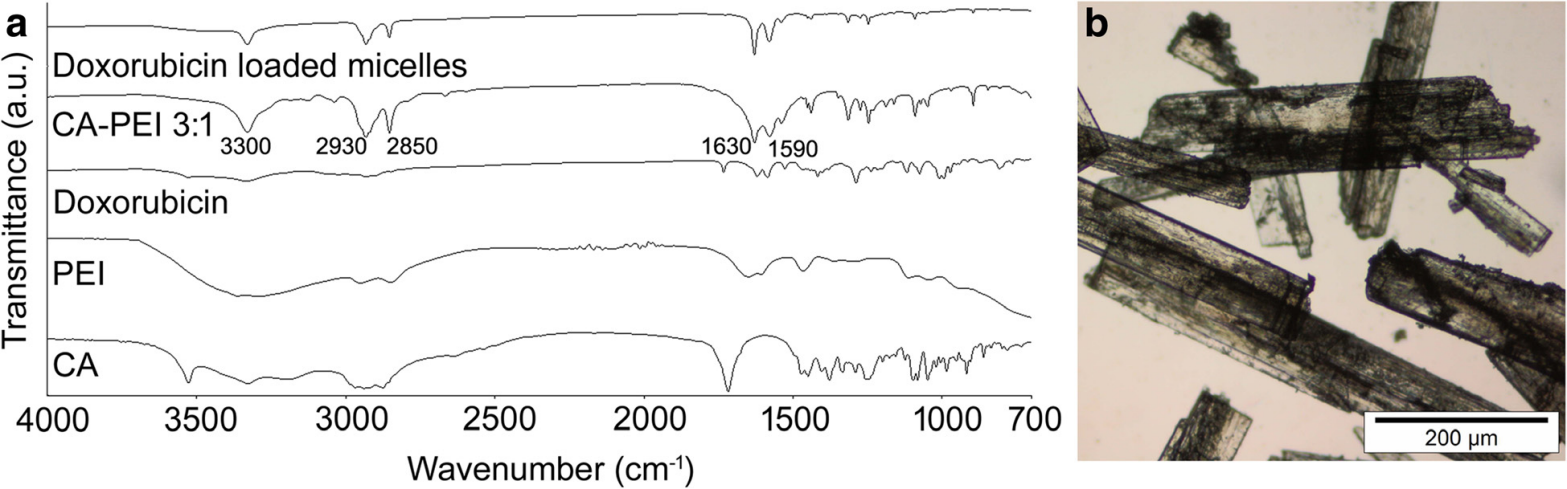
**FTIR spectra and light microscope image.** FTIR spectra of CA, PEI, doxorubicin, CA-PEI 3:1 blank micelles, and doxorubicin-loaded CA-PEI 3:1 micelles (**a**). Light microscope image of CA-PEI 3:1 micelles (**b**).

The FTIR spectra of the conjugates were somewhat consistent between the molar ratios tested (1:1, 1:2, 1:4, 3:1, and 4:1) (Figure 2a). In the CA-PEI spectra, peaks for the N-H bending, C = O absorbance band, and C-H and N-H stretching were observed at 1,590, 1,630, 2,850 to 2,930, and 3,300 cm^−1^, respectively. The overlapping of the C = O absorbance band (1,630 cm^−1^) with the N-H bending band (1,590 cm^−1^) appeared as a doublet in the CA-PEI spectra. This indicated the formation of an amide linkage between CA and PEI [17]. The spectra of the doxorubicin-loaded micelles indicated the absence of the characteristic peaks for doxorubicin, showing that the drug was contained within the hydrophobic micelle core [18].

The freeze-drying process produced white crystalline CA-PEI conjugates where their morphology was observed under the light microscope as shown in Figure 2b. The synthesized conjugates appeared as slender, needle-shaped small units. Each unit could be distinguished separately, and the length of the units varied slightly.

**Figure 3:**
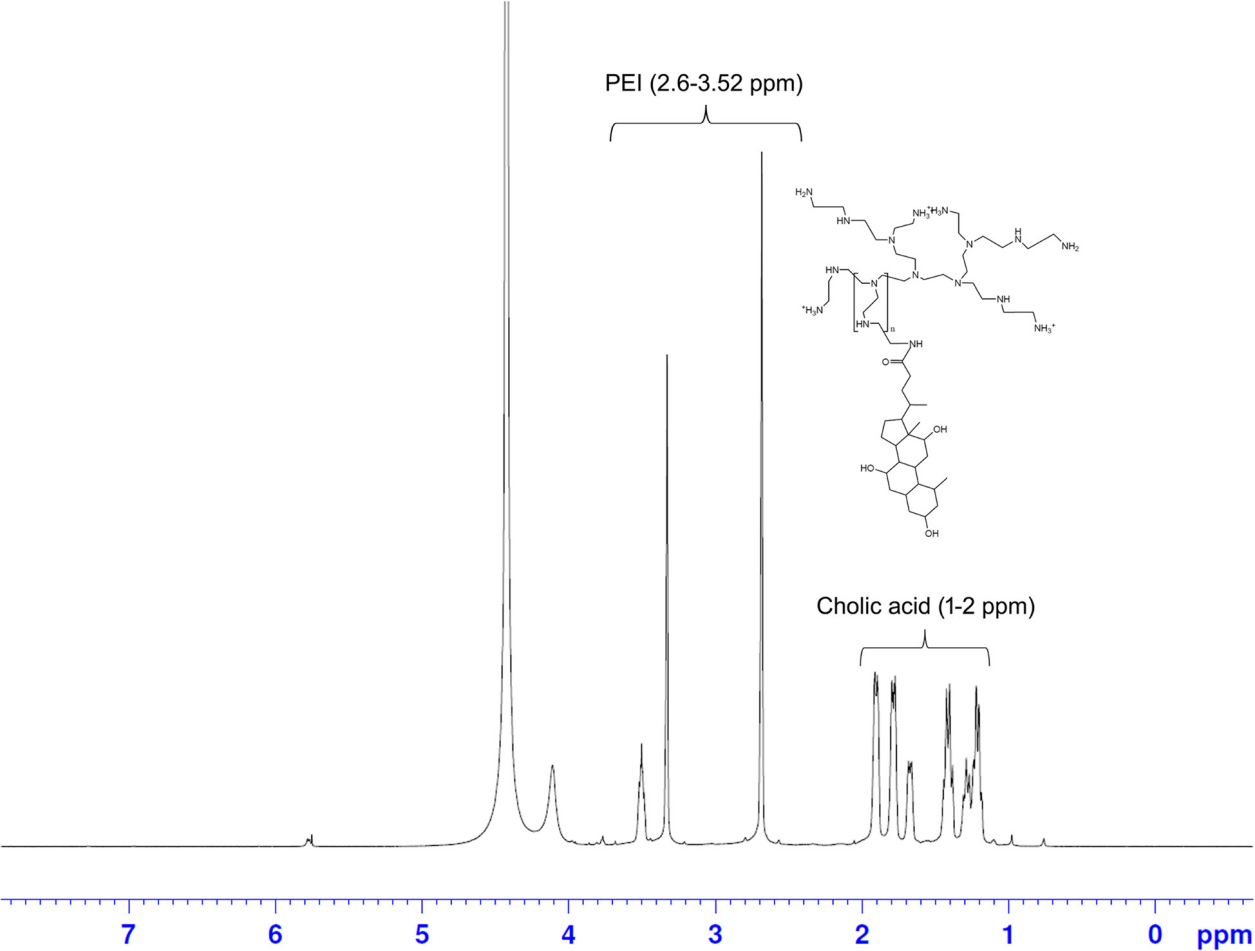
^**1**^**HNMR spectrum of CA-PEI copolymer at a molar feed ratio of 3:1.**

In the hydrogen nuclear magnetic resonance (^1^HNMR) spectra (Figure 3), proton shifts were observed in the region of 1 to 2 ppm, which are the characteristic peaks of CA. These are the doublet, triplet, and multiplet peaks indicating the structure of CA. Integration values in the region of 1 to 2 ppm designate the number of protons in CA. Proton shifts from 2.6 to 3.52 ppm indicated the presence of PEI. At 4.5 ppm, there was a proton shift of the solvent.

**Figure 4:**
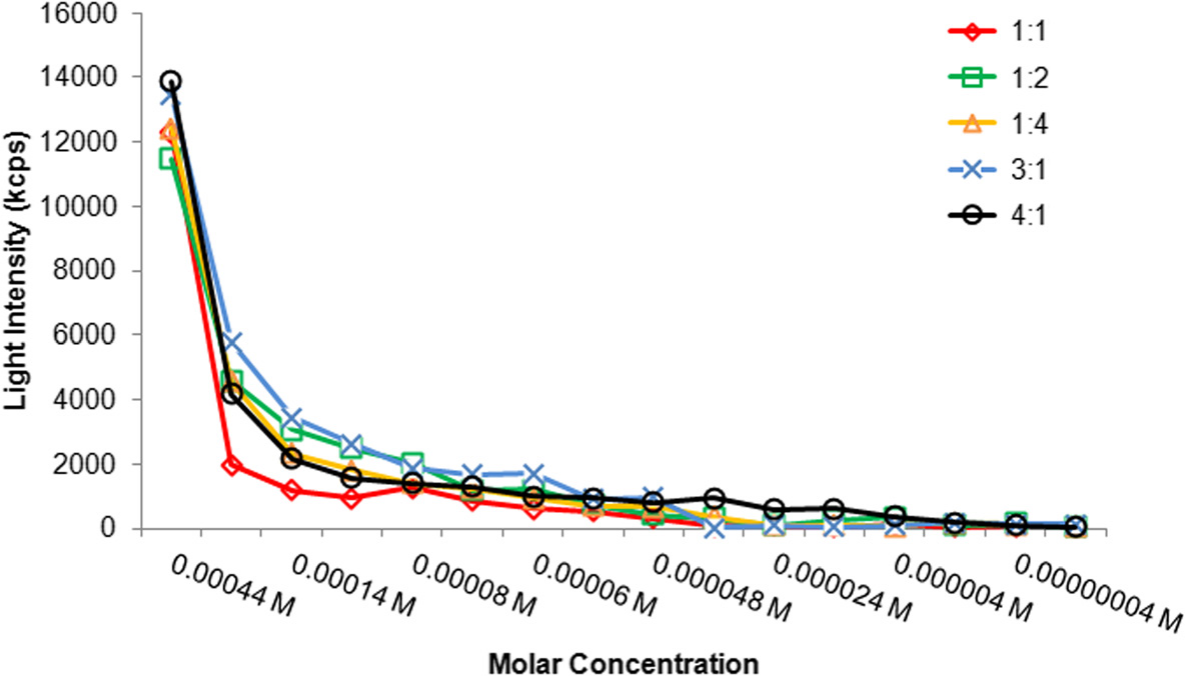
Critical micelle concentrations of CA-PEI micelles.

The CMCs of a series of CA-PEI solutions of different molar ratios are shown in Figure 4. Changes in the light intensity are symbolized as a function of the molar concentration, in which an abrupt increase designates the formation of stable micelles. The results showed that the micelles at 3:1 ratio had a lower CMC than those at other ratios. Given that CA has a hydrophobic steroidal nucleus, an increase in CA units could add to the hydrophobic interactions between the polymer chains in the micelle core and stabilize the structure. This is significant for the drug solubilization and EE of micelles [19,20]. However, the CA-PEI micelles were ideally stable merely up to a definite concentration of CA (3:1). When the molar fraction of CA was raised further, it also increased the hydrophilic segments, which raised the likelihood of interaction between the hydrophilic and hydrophobic segments and a decreased hydrophobicity of the core, consequently leading to an increased CMC.

High CMCs are a key problem linked to micelle formulations given intravenously or diluted in blood. Low CMCs of CA-PEI micelles would thus offer some benefits, such as stability against dissociation and precipitation in blood due to dilution. In addition, embolism caused by the elevated amount of polymers used for the micelle formation could be avoided [21].

**Figure 5:**
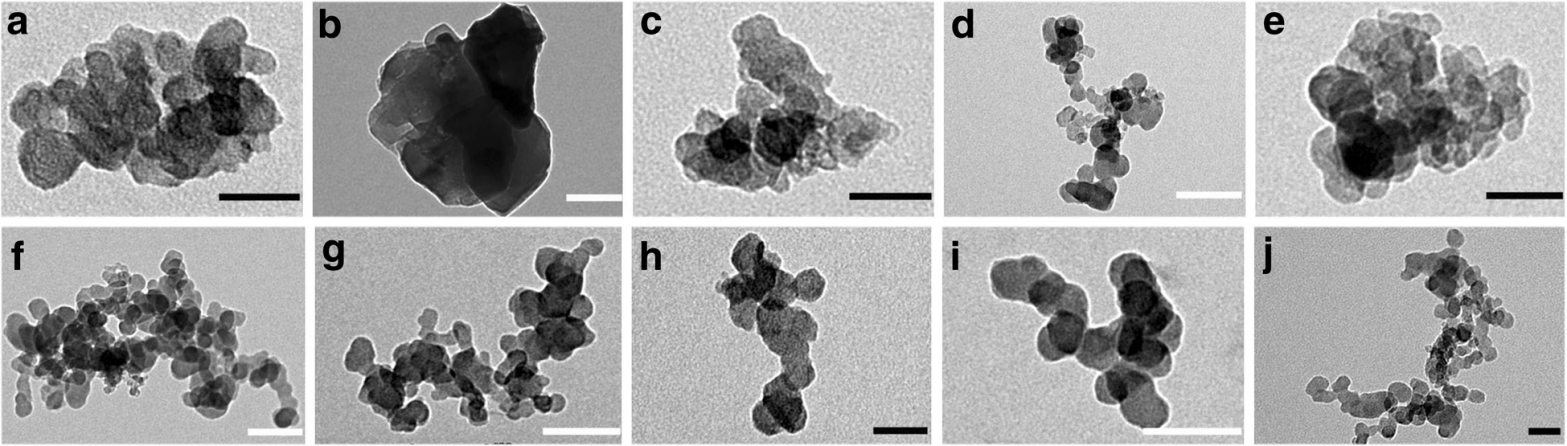
**TEM images of CA-PEI micelles.** CA-PEI 3:1 (**a**, **b**), CA-PEI 1:1 (**c**, **d**), CA-PEI 4:1 (**e**, **f**), CA-PEI 1:2 (**g**, **h**), and CA-PEI 1:4 (**i**, **j**). Black scale bars represent 100 nm, and white scale bars represent 50 nm. The magnification of the images were × 160,000 (**a**, **c**), ×135,000 (**e**, **h**, **j**), ×105,000 (**b**, **d**, **i**), and × 87,000 (**f**, **g**).

TEM micrographs of the CA-PEI micelles are shown in Figure 5. The micelles were observed to have a spherical shape and were uniform in size ranging from 150 to 200 nm. The bright areas perhaps encompassed the hydrophobic part forming the micellar core, whereas the hydrophilic corona appeared to be darker because this region has a higher electron density than the core [22].

**Figure 6:**
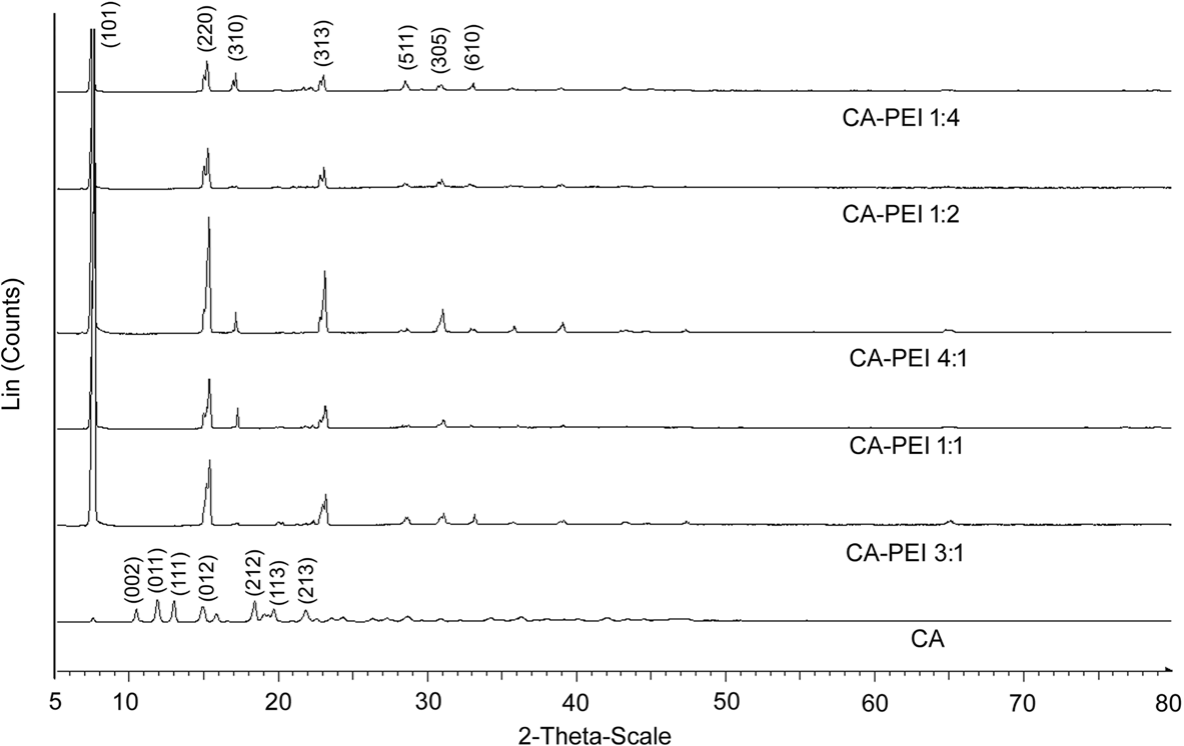
XRD patterns of CA and CA-PEI conjugates of five different molar feed ratios.

The formation of small, lustrous CA-PEI conjugates (1 to 2 mm) was an interesting finding; hence, they were subjected to XRD analysis (Figure 6). For CA alone, characteristic peaks were observed at 2*θ* = 12.0°, 13.1°, and 19.8° [23]. In contrast, the XRD patterns of the CA-PEI conjugates showed characteristic body-centered lattice peaks at 2*θ* = 7.6°, 15°, and 23.2°. The intensity of the peak at 2*θ* = 7.6° was maximum for all CA-PEI conjugates. The sharp, intense, and broad peaks of the CA-PEI conjugates indicated a crystalline nature of the conjugate.

**Figure 7:**
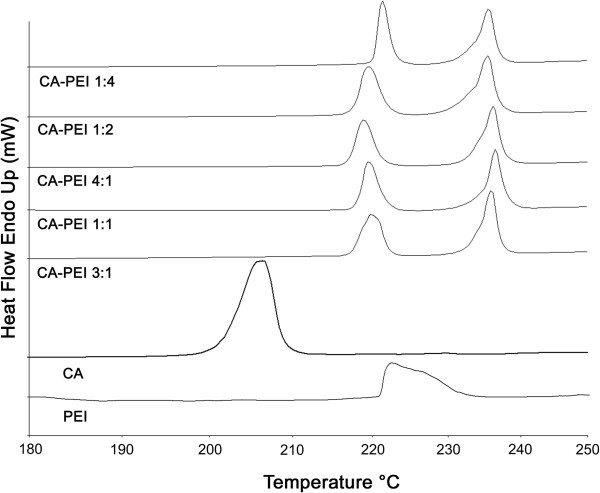
DSC curves of CA, PEI, and CA-PEI conjugates with five different molar feed ratios.

The conjugates were then subjected to DSC analysis (Figure 7). When heated from 30°C to 250°C at 20°C/min, the CA crystals exhibited endothermic peaks due to fusion at 202°C [24], while a broad endothermic peak of a relatively lesser intensity was observed for PEI at 220°C. The DSC curve of the CA-PEI conjugate had two fusion peaks derived from CA and PEI at 220°C and 235°C, indicating the formation of conjugates. The intensity of the first peak was slightly higher than that of the second peak.

**Figure 8:**
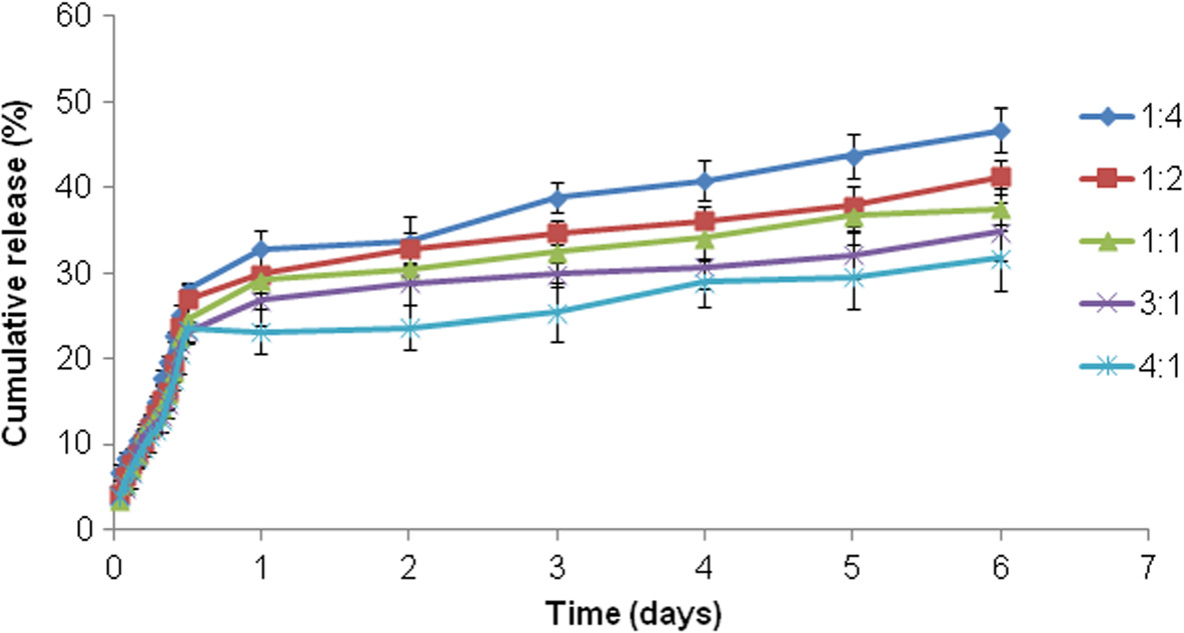
Doxorubicin release from CA-PEI micelles at pH 7.4.

DLC and EE of micelles as calculated using Equations 1 and 2 are represented in Table 1. The *in vitro* release profile of the doxorubicin-loaded micelles in PBS solution (pH 7.4) was obtained, which is summarized in Figure 8. The drug release decreased as the drug content increased in the micelles. Micelles with a molar ratio (CA-PEI) of 1:4 had the maximum doxorubicin release after 6 days. The micelles exhibited a sustained release pattern of doxorubicin, which was characterized by an initial burst release followed by a slow and continuous drug release. In fact, this is a frequent observation for doxorubicin release reported by a number of researchers [25-29]. Doxorubicin is recognized to form a dimer in aqueous media due to the chemical reaction between the 30-NH_2_ group and the C9 α-ketol side chain. Given that the doxorubicin dimer is almost water insoluble and that its azomethine bond may readily be cleaved to restore the doxorubicin monomer, the later stage of sustained drug release may involve regenerated doxorubicin in addition to the doxorubicin dimer itself [30].

**Table 1:** DLC and EE of doxorubicin-loaded micelles

**CA/PEI**	**DLC (% *w/w*)**	**EE (% *w/w*)**
1:4	0.89	56.52
1:2	0.96	59.44
1:1	1.06	61.31
3:1	1.28	67.57
4:1	1.19	64.22

### *In vitro* cell cytotoxicity

**Figure 9:**
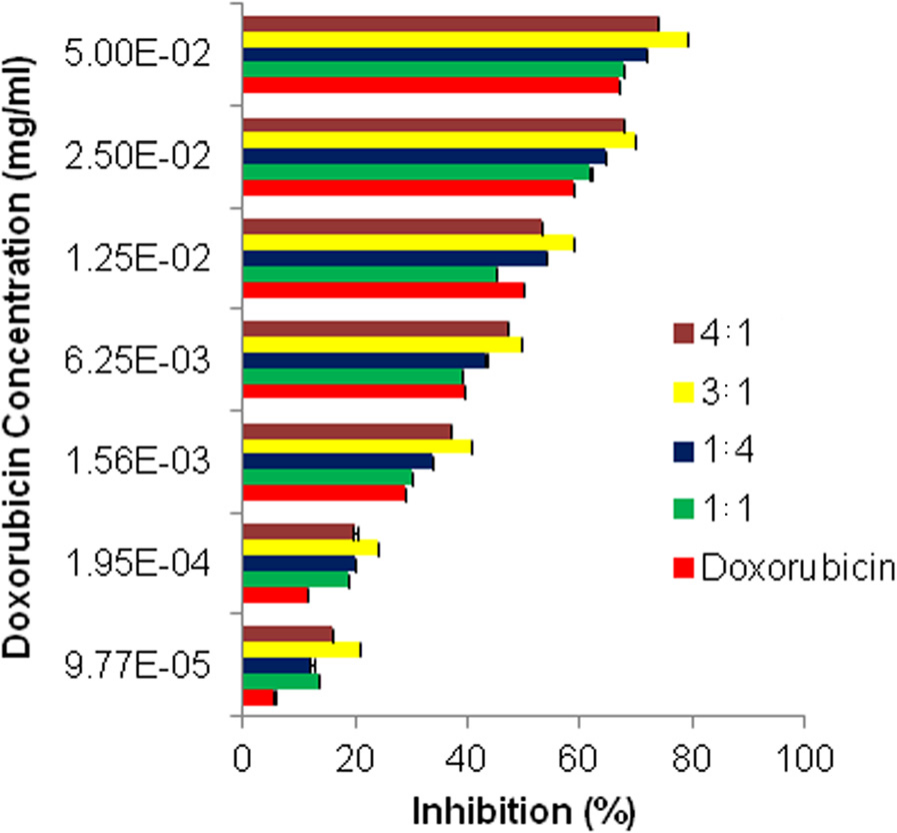
**Cytotoxicity of doxorubicin-loaded micelles on DLD-1 cells after 24 h.** Twenty thousand cells were exposed to doxorubicin and doxorubicin-incorporated CA-PEI micelles for 24 h.

**Figure 10:**
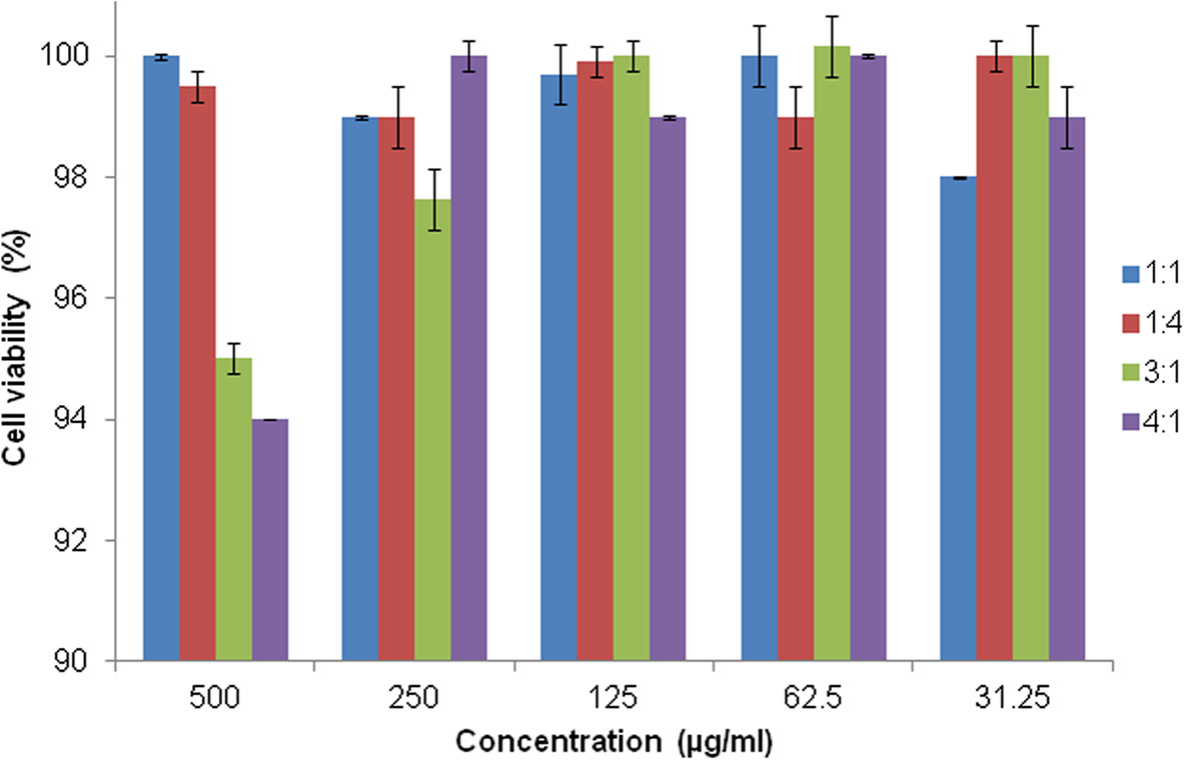
Cell viability (%) of V79 cells at 24 h post-incubation with increasing concentrations of CA-PEI blank micelles.

As shown in Figure 9, the percent inhibition of cancer cells by the doxorubicin-loaded micelles improved from the 1:4 to the 4:1 combinations. Incorporation of doxorubicin into the CA-PEI micelles increased its cytotoxicity toward cancer cells. The half-maximal inhibitory concentration (IC_50_) values for the doxorubicin-loaded micelles were lower than those for free doxorubicin. The lower percentage inhibition and superior IC_50_ of doxorubicin compared with those of the doxorubicin-loaded micelles may well be accredited to the formation of aggregates, which deter drug entry into the cells. In addition, doxorubicin could be removed from tumor sites by drug efflux pumps [31]. In contrast, the enhanced cytotoxicity of the doxorubicin-loaded micelles could be explained by the higher permeability and retention of micelles in tumor cells. In addition, increased penetration of the doxorubicin-loaded micelles makes it possible for the drug to be delivered to the site of action, which is located in the nucleus, and therefore gives more time for doxorubicin to interact with its substrate. The increased cytotoxicity observed toward cancer cells could be linked to an increased production of reactive oxygen species and enhanced apoptosis. The ability of CA to modulate the number of aberrant crypt foci by restraining their development and growth and by eliminating a selected population may also contribute to the cytotoxicity of the doxorubicin-loaded micelles [32]. Both free doxorubicin and entrapped doxorubicin caused cell death in a dose-dependent manner. The cytotoxicity of doxorubicin is likely to increase further *in vivo* due to the enhanced permeation and retention effects of the loaded micelles. These findings imply that the selective uptake of micelles by cancer cells could reduce the toxicity and adverse effects of doxorubicin. To verify the low toxicity of blank micelles to normal cells, cell viability was determined in V79 cells (Figure 10). The blank micelles were not toxic to V79 cells in the tested concentration ranges.

## Conclusions

Here, we report the synthesis of doxorubicin-loaded novel CA-PEI micelles for the first time. The conjugates readily formed micelles, which exhibited a uniform spherical morphology as observed by TEM. XRD analysis revealed that the conjugates had a crystalline structure. Increasing the quantity of incorporated doxorubicin decreased the release rate of the drug. Doxorubicin-loaded CA-PEI micelles had an enhanced antitumor activity against tumor cells *in vitro* compared with that of doxorubicin itself. In contrast, when blank micelles were exposed to normal (V79) cells, they did not exhibit considerable toxicity. Together, these results indicate the potential of doxorubicin-loaded CA-PEI micelles as carriers for targeted antitumor drug delivery system.

## Abbreviations

CA: Cholic acid; CMC: Critical micelle concentration; DCC: Dicyclohexylcarbodiimide; DLC: Drug loading content; DMEM: Dulbecco’s modified Eagle’s medium; DSC: Differential scanning calorimetry; EE: Entrapment efficiency; FTIR: Fourier transform infrared; HNMR: Hydrogen nuclear magnetic resonance; MW: Molecular weight; MWCO: Molecular weight cutoff; NHS: *N*-hydroxysuccinimide; PBS: Phosphate-buffered saline; PEI: Polyethyleneimine; TEM: Transmission electron microscopy; XRD: X-ray diffraction.

## Competing interests

The authors declare that they have no competing interests.

## Authors’ contributions

MWA carried out the preparation, characterization, drug loading, and drug release studies of cholic acid-polyethyleneimine micelles. HK and AMB participated in the cell viability assays. MCIMA participated in the design of the study and coordination. MWA and AMB drafted the manuscript. All authors read and approved the final manuscript.
